# Comprehensive Review of Molecular Mechanisms during Cholestatic Liver Injury and Cholangiocarcinoma

**DOI:** 10.4172/2167-0889.1000231

**Published:** 2018-09-20

**Authors:** Shohaib Virani, Austin Akers, Kristen Stephenson, Steven Smith, Lindsey Kennedy, Gianfranco Alpini, Heather Francis

**Affiliations:** 1 Research, Central Texas Veterans Health Care System, Texas, USA; 2 Department of Internal Medicine, Baylor Scott & White Health, Texas, USA; 3 Department of Medical Physiology, College of Medicine Texas A&M Health Science Center, Temple, Texas, USA

**Keywords:** Cholangiopathies, Therapy, Molecular mechanisms

## Abstract

Cholestatic liver injury is characterized by damage induced on the biliary tree and cholangiocytes, the cells lining the biliary tree, thus they are termed “cholangiopathies”. Cholangiopathies include diseases such as Primary Biliary Cholangitis, Primary Sclerosing Cholangitis, Biliary Atresia and Cholangiocarcinoma. These pathologies lack viable therapies and most patients are diagnosed during late stage disease progression (with the exception of Biliary Atresia, which is found shortly after birth). The lack of therapies for these diseases has put a significant burden on the need for liver transplantation as this is the only indicative “cure” for cholangiopathies. The molecular mechanisms for cholangiopathies have been extensively studied; however, and unfortunately, the lack of effective biomarkers and therapeutics remains. In this review article we highlight the latest studies to investigate the molecular mechanisms regulating cholangiopathies and the potential therapeutics that might be discovered.

## Primary Biliary Cholangitis

### Introduction

**Figure 1:**
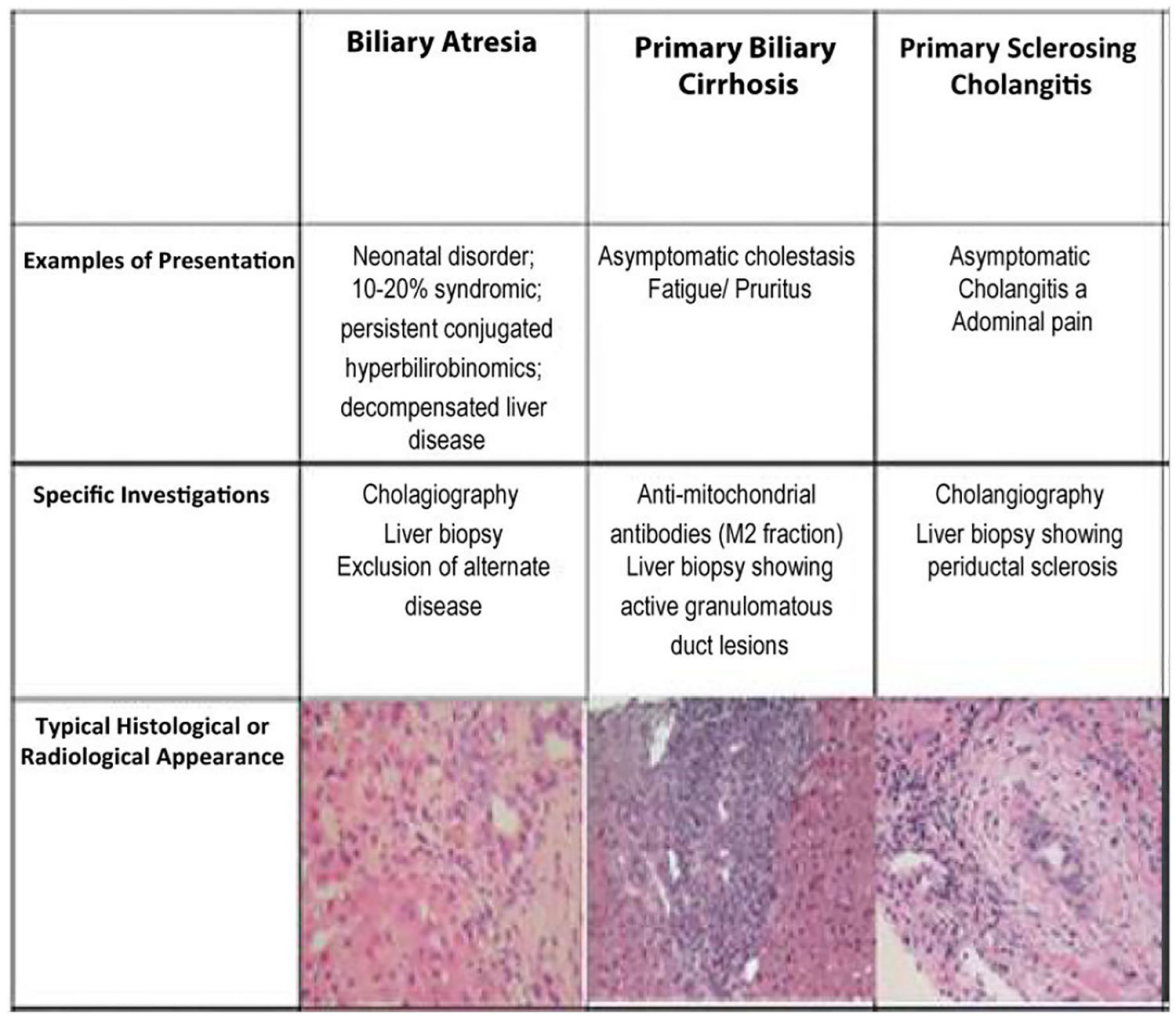
Presentation, investigation and typical histological appearance of BA, PBC and PSC. Modified and reprinted with permission from: Hirschfield G.M. (2014) The Diagnosis and Classification of Immune-Mediated Biliary Diseases. In: Gershwin M., Vierling J., Manns M. (eds) Liver Immunology. Springer, Cham.

Initially described in 1851, primary biliary cirrhosis, now known as primary biliary cholangitis (PBC), is a chronic autoimmune disease seen primarily in middle-aged women [1]. PBC results in progressive destruction of intrahepatic bile ducts causing subsequent chronic cholestasis, portal inflammation, and fibrosis, which can ultimately lead to cirrhosis and hepatic failure [2]. The clinical presentation of PBC is comprised primarily of pruritis, fatigue and other generalized symptoms. Ultimately, the diagnosis is based on two out of the three following criteria: the presence of biochemical cholestasis (an elevated ALP), presence of ant-mitochondrial antibodies (AMA), and classic histological findings of PBC on a liver biopsy (destructive cholangitis affecting the interlobular bile ducts) [3,4]. In a T2-weighted MRI, the classical presentation of a liver with PBC would show fibrosis of the parenchyma of the liver with a periportal halo sign. Other signs could include periportal hypersensitivity, regional lymphadenopathy and splenomegaly [5]. The prevalence of PBC ranges between 6.7 and 402 cases per million persons, while the annual incidence ranges between0.7 and 49 cases per million persons [6]. Patients with PBC typically range in presentation from asymptomatic to fulminant liver failure necessitating liver transplant [7]. Most commonly, patients present with pruritus and fatigue as well as jaundice, xanthomas, osteoporosis, and dyslipidemia [8]. PBC affects approximately 9 females to 1 male and is multifactorial in etiology including genetic predisposition and environmental insults [9]. Elevated anti-mitochondrial antibody (AMA) is the hallmark of the disease and is present in greater than 90% of individuals with the condition [1]. AMA has replaced the need for liver biopsy in order to make a diagnosis of PBC [7]. Despite major medical advances, there remains only one FDA approved medication for the treatment of PBC, ursodeoxycholic acid (UDCA) [1]. UDCA is typically prescribed at a dose of 13–15 mg/kg body weight. Higher doses in patients with inadequate response have shown to be ineffective and, in a few cases, higher doses may be more damaging [1,7]. Studies have demonstrated improved transplant-free survival in patients on UDCA monitored at one year with reduction of alkaline phosphatase (ALP) and aspartate transaminase (AST) to <1.5 times the upper limit of normal, and a normal total bilirubin after one year of treatment [1]. Figure 1 describes the presentation, investigation and histological appearance of PBC.

### Molecular mechanism of injury

PBC is largely suspected to be secondary to an autoimmune process involving the liver, which leads to destruction of small and mediumsized bile ducts. Histological findings include damaged cholangiocytes and infiltration of the portal area with B cells, T cells, macrophages, eosinophils, and natural killer (NK) cells increasing the risk for cirrhosis and hepatic failure [6]. Sjogren syndrome, autoimmune thyroid disorders, and Raynaud syndrome as well as urinary tract infections have been linked with PBC. An epidemiological study showed that 48% of PBC patients experienced prior recurrent urinary tract infections versus 31% of controls [10]. Most commonly, urinary tract infections in women are caused by Escherichia coli (*E. coli*) prompting its study in relation to PBC. *E. coli* infections are suspected to cause molecular mimicry of *E. coli* proteins with human dehydrogenase complex leading to the induction of B- and T-cell responses and subsequent autoimmune responses typically found in PBC [8].

Animal models have demonstrated that the etiology of PBC is multifactorial requiring both environmental insults and genetic predisposition in order to break tolerance and lead to eventual liver pathology [9]. These models have shown that NKT cells play an important role in the initiation of disease in the serologically positive patients, while CD8^+^ memory T cells are directly involved in the destruction of cholangiocytes [9].

Reactive oxygen species are produced via many microscopic processes that take place within the cells [11,12]. These reactive molecules induce DNA damage and initiate protein degradation [11]. Nitric oxide-derived oxidative kills pathogens, mediate the immune response, and lead to cellular damage [13]. Nitric oxide-derived oxidative species lead to prolonged interruption of the circulation of bile acids, ultimately leading to cholestasis and the induction of PBC [14].

### New therapeutic targets

UDCA has not been shown to improve all-cause mortality, pruritis, fatigue, or outcomes from liver transplantation, but it was found to have a beneficial effect on the histological examination [15]. For patients who do not respond to UDCA, there are currently no alternative treatments that delay the progression of PBC [1]. Liver transplant continues to be the definitive treatment for advanced disease, with an approximate 70%, 10-year survival following transplantation [8]. Post-transplant recurrence occurs in approximately 30% at 10 years and 40% at 15 years, which is often a challenging diagnosis given that AMA remains persistently positive in most patients [8].

Obetocholic acid is a farsenoid-X-receptor agonist that is currently being studied when given in addition to UDCA. The semi-synthetic analogue of chenodeoxycholic acid is present in the liver, kidneys, adrenal glands, and intestine. It exerts its action on 7 alpha hydroxylase leading to decreased bile acid uptake proteins and increased expression of bilirubin exporter pumps, thereby reducing bile acid synthesis and reducing their toxic effects [1,7,8].

Pruritis is the predominant complaint of patients suffering from cholestatic liver disease [16]. Cholestatic itch has long been countered by the cholestyramine treatment, which works by reducing bile acid reabsorption [17]. For patients who do not respond to cholestyramine or who cannot tolerate its side effects, plasmapharesis remains an option [7]. Previous studies have shown that plasmapharesis is a method for treating refractory pruritus but further studies are needed to establish when this option should be utilized [7].

Umbilical cord-derived mesenchymal stem cell (UC-MSC) transfusion has been studied in order to delay or prevent PBC progression in patients who did not respond to UDCA. It is thought that UC-MSC may suppress the antigen-induced autoimmune condition, as well as stimulate repair of the injured bile ducts. In addition, US-MSC was shown to improve quality of life in PBC patients as it alleviated pruritus and fatigue [18].

### Future outlook on the disease

Current studies show that indoleamine 2,3 dioxygenase (IDO) could play a potential role in PBC. IDO is an intracellular enzyme, which functions as an immunosuppressant. It is thought that impaired IDO expression is involved in the progress of autoimmunity in PBC. The effects that IDO exerts on tryptophan and its catabolism could contribute to potential treatment opportunities and biomarkers for disease progression [19].

Liver stiffness measurement (LSM) is currently being studied, as there are no reliable markers of liver fibrosis in PBC. Transient elastography (TE) was studied in a large cohort to monitor UDCA-treated patients and noninvasively assess liver stiffness. As there are no current serum surrogate markers of liver fibrosis routinely monitored in those with PBC, LSM could play an important role for clinicians to evaluate treatment. Monitoring of TE provided important prognostic information for PBC patients, in particular, those with cirrhosis and may be of benefit to predict outcomes. It was also associated with elevated levels of hyaluronic acid, which may be useful to monitor in PBC patients. Confirmation of these results is needed with larger studies, though improved monitoring in regards to response to treatment may be on the horizon [20].

Levels of albumin, ALP, and bilirubin have been studied as surrogate endpoints in therapy trials. Survival of patients with advanced disease and biochemical response to UDCA has significantly improved for those without response. There is discussion whether albumin and bilirubin are superior to the information obtained from ALP. In addition, questions remain regarding the appropriate time to monitor for improvement of these markers at either 6 months or 1 year [21,22].

There are several promising alternatives such as obetocholic acid, which are still undergoing trials. Appropriate monitoring concerning response to treatment also has questions to be answered as to whether liver function tests such as ALP, albumin, and bilirubin are as accurate as TE. Ideally, future studies will continue to solve the mystery of PBC’s mechanism of action and identify new information pertaining to IDO that may play a role in treatment and possibly decrease the number transplants. Post-transplant recurrence requires additional studies, as there is no current guideline directed therapy for these patients.

## Primary Sclerosing Cholangitis

### Introduction

Primary sclerosing cholangitis (PSC) involves chronic inflammation, fibrosis, and eventual destruction of the intra- and extra-hepatic biliary system [23,24]. PSC is typically diagnosed in the third through fifth decades of life and has an approximately 3 to 1 male to female predilection. Diagnosis of PSC after the age of 50 is considered late onset and one study showed that late onset PSC was associated with more frequent dilatation therapy, recurrent cholangitis, and decreased transplant-free survival when compared to those diagnosed with PSC before age 50 [25]. PSC is a relatively rare disease and while the incidence is not well established, a systematic review of 8 studies from North America and Europe in 2011 showed an overall incidence of 0.77 per 100,000 person years [26]. The prevalence data has varied and even the data on the higher end of the spectrum reveals a prevalence of only 6 per 100,000 individuals [27]. Figure 1 describes the presentation, investigation and histological appearance of PSC.

There is a strong association between PSC and inflammatory bowel disease (IBD), particularly ulcerative colitis (UC). Studies have consistently shown that greater than 70% of PSC patients have concurrent IBD, the vast majority of which will be UC, and 5–7.5% of UC patients will eventually be diagnosed with PSC. Thus, patients diagnosed with PSC are recommended to routinely undergo screening for IBD. Individuals with PSC and IBD have been shown to have a significantly higher probability of developing colorectal cancer or cholangiocarcinoma [28,29]. One study showed that patients with both PSC and IBD carried a two-fold increased risk of developing advanced colorectal neoplasia compared to patients with only IBD [29]. Furthermore, PSC is the most significant known risk factor for cholangiocarcinoma and these patients carry a lifetime risk of 10 to 15 percent [30–32]. PSC is also a risk factor for other hepatobiliary cancers including hepatocellular carcinoma and gallbladder carcinoma. Ali et al. demonstrated that approximately 9.5% of patients with PSC over a 20-year time period developed hepatobiliary cancer [33]. Of those, 78% developed cholangiocarcinoma, 21% developed hepatocellular carcinoma, and 6% developed gallbladder carcinoma [33]. Given the risk of developing hepatobiliary cancer, the AASLD recommends surveillance for hepatobiliary cancer with an annual ultrasound. One study showed that patients with PSC who underwent surveillance for hepatobiliary cancer had significantly higher 5-year survival (68% vs. 20%, respectively) [33]. Survival times vary considerably based on the stage of disease at the time of diagnosis, with one study showing a median survival time from diagnosis to either death or liver transplantation of 9.6 years [30].

About half of the patients suffering from PSC are asymptomatic at the time of diagnosis, despite having advanced stages of the disease. The most common symptoms that symptomatic patients present with include fatigue and pruritis [34,35]. Elevated liver enzymes in an obstructive pattern are the most common laboratory finding, with a predominantly elevated ALP level [36]. A large portion of PSC patients are positive for p-ANCA, anti-SM and ANA antibodies, however these antibodies have low specificity for PSC and frequencies vary between studies [37]. PSC can be diagnosed on cholangiography with findings of bile duct strictures with segmental dilation, giving the appearance of “beading” [38]. Cholangiography is obtained via magnetic resonance cholangiopancreatography (MRCP), endoscopic retrograde cholangiopancreatography (ERCP), or percutaneous trans-hepatic cholangiography (PTC) [39]. As the least invasive method, MRCP carries minimal risk [36,39]. Liver biopsy is usually unnecessary for diagnosis and its utility in the evaluation of liver disease is declining [36,39].

There is currently no FDA approved pharmaceutical treatment for PSC. UDCA has been the most extensively studied treatment, and although it has been shown to reduce ALP levels, evidence of clinical improvement and prolonged survival is lacking. Lindor et al. showed that UDCA at doses of 13 to 15 mg per kg of body weight per day resulted in improvement in ALP, AST, bilirubin, and albumin levels but no significant difference in time to treatment failure or time to liver transplantation [40]. This study further showed that serious adverse effects including death, the development of varices, and liver transplant eligibility occurred more often in the group treated with UDCA [41]. Additionally, meta-analysis by Triantos et al. showed that when comparing standard or high dose UDCA to placebo or no intervention there was no significant difference outcome in regards to symptoms or progression of disease [42]. Given the lack of clinical efficacy and potential for adverse effects, the use of UDCA remains controversial. ACG guidelines from 2015 do not recommend for or against using UDCA in the treatment of PSC.

Medical therapy of PSC that involves altering the gut microbiota is of increased interest. One case report showed that four weeks of daily fecal microbiota transplant enemas in a patient with UC and PSC resulted in improvement of IBD symptoms and normalization of liver enzymes [43]. Further research is needed to determine whether normalization of liver enzymes correlates with clinical improvement. Vancomycin has been studied in PSC treatment and a case study showed a 12-year-old female with PSC status post liver transplant was treated with oral vancomycin 500 mg three times daily [44]. With this treatment, liver enzymes normalized and repeat liver biopsy with three years of continued treatment showed no evidence of bile duct inflammation [44].

The only definitive treatment of PSC is liver transplantation; however, this is only indicated in a subset of patients. Indications include recurrent bacterial cholangitis, decompensated cirrhosis, hepatocellular carcinoma, hilar cholangiocarcinoma and sequelae of portal hypertension. Among patients who receive liver transplantion, clinical recurrence after transplant remains a possibility. A systematic review by Gautam et al. showed a recurrence rate after liver transplant of 11 percent [45]. Another study showed that over time, risk of recurrence increases from 2% at 1 year to as high as 20% at 10 years, and those who were diagnosed with cholangiocarcinoma prior to initial liver transplant carried a significantly higher risk of recurrence[46].

### Molecular mechanisms of injury

The pathological mechanisms of PSC are incompletely understood and likely multifactorial. Intestinal microbial dysbiosis is one proposed mechanism, given the poorer outcomes (shorter transplant-free and overall survival) and higher rates of cholangiocarcinoma amongst those with concurrent IBD and PSC [47]. The translocation of intestinal bacteria across an inflamed, permeable gut combined with decreased proliferation of other beneficial organisms may result in an overall increased immune response to intestinal endotoxins [48,49].

The concept of cellular senescence and the senescence-associated secretory phenotype (SASP) is an emerging mechanism of cholangiocyte damage and destruction in PSC [50]. This phenotype is associated with increased pro-inflammatory chemokines, cytokines, and extracellular matrix remodeling proteases. Overall, PSC cholangiocytes differ significantly from normal human cholangiocytes. They exhibit features such as “diminished monolayer formation, barrier function, and associated tight junction protein expression, as well as impaired growth and upregulation of inflammatory signaling molecules,” as demonstrated in a 2014 study which characterized cultured PSC cholangiocytes [51]. SASP is linked with dysbiosis, as UDCA is a commensal gut bacteria metabolite which abrogates cholangiocyte senescence in vitro and, when absent in animal models, results in cellular senescence and exacerbated biochemical and histological features of PSC [51].

Additionally, disruptions in biliary epithelial cell tight junctions may expose cholangiocytes to a variety of substances, which can activate an inflammatory response. These disruptions are mediated by toll-like receptor (TLR) mechanisms and inflammation is induced by a variety of molecular mediators, which ultimately leads to myofibroblast activation and fibrosis. As discussed in a 2013 article, in liver explants from patients with PSC, biliary epithelial cells express higher levels of TLR, nucleotide-binding oligomerization domain, the MyD88/IRAK complex, tumor necrosis factor (TNF)α, interferon (INF)γ, and IL8, than cells from individuals without PSC [52].

### New therapeutic targets

N-Ras is a known inducer of cellular senescence and is increased in PSC cholangiocytes 50. Additionally, experimentally induced senescent human cholangiocytes have been shown to cause senescence in bystander cholangiocytes. Targeted therapy towards this protein and other inducers of cellular senescence could have significant utility in the treatment of PSC.

One of the proposed mechanisms by which PSC and IBD are linked is the upregulation of vascular adhesion protein 1 (VAP-1) on mesenteric vessels. VAP-1 induces the generation of mucosal addressin cell adhesion molecule 1 (MAdCAM-1) and the chemokine CCL25 in the gut and on liver sinusoidal endothelium. The expression of these adhesion molecules and chemokines is increased in the PSC liver as well. Ultimately this results in the increased lymphocyte recruitment to the liver and biliary tree, resulting in inflammation and cellular destruction [53]. Agents targeting these and other adhesion molecule/chemokine interactions are currently undergoing trials for the treatment of IBD and may show future utility in PSC treatment.

Existing evidence in animal models suggests that 24-*nor*UDCA, a C23 homologue of UDCA, is superior to UDCA in improving AP and ALT levels, markers of inflammation, and liver histology [53]. As it is primarily secreted in the unchanged glucuronidated form, 24-*nor*UDCA increases hydrophilicity of biliary bile acids stimulating bile flow. This increased elimination decreases cholangiocyte inflammatory response. Phase II clinical trials in humans have yielded favorable results, with evidence that 24-*nor*UDCA dramatically decreases ALP levels in a dose-dependent manner [54]. Additionally, it has been shown to have a favourable safety profile with minimal side effects, and these results warrant further evaluation with phase III trials.

**Figure 2:**
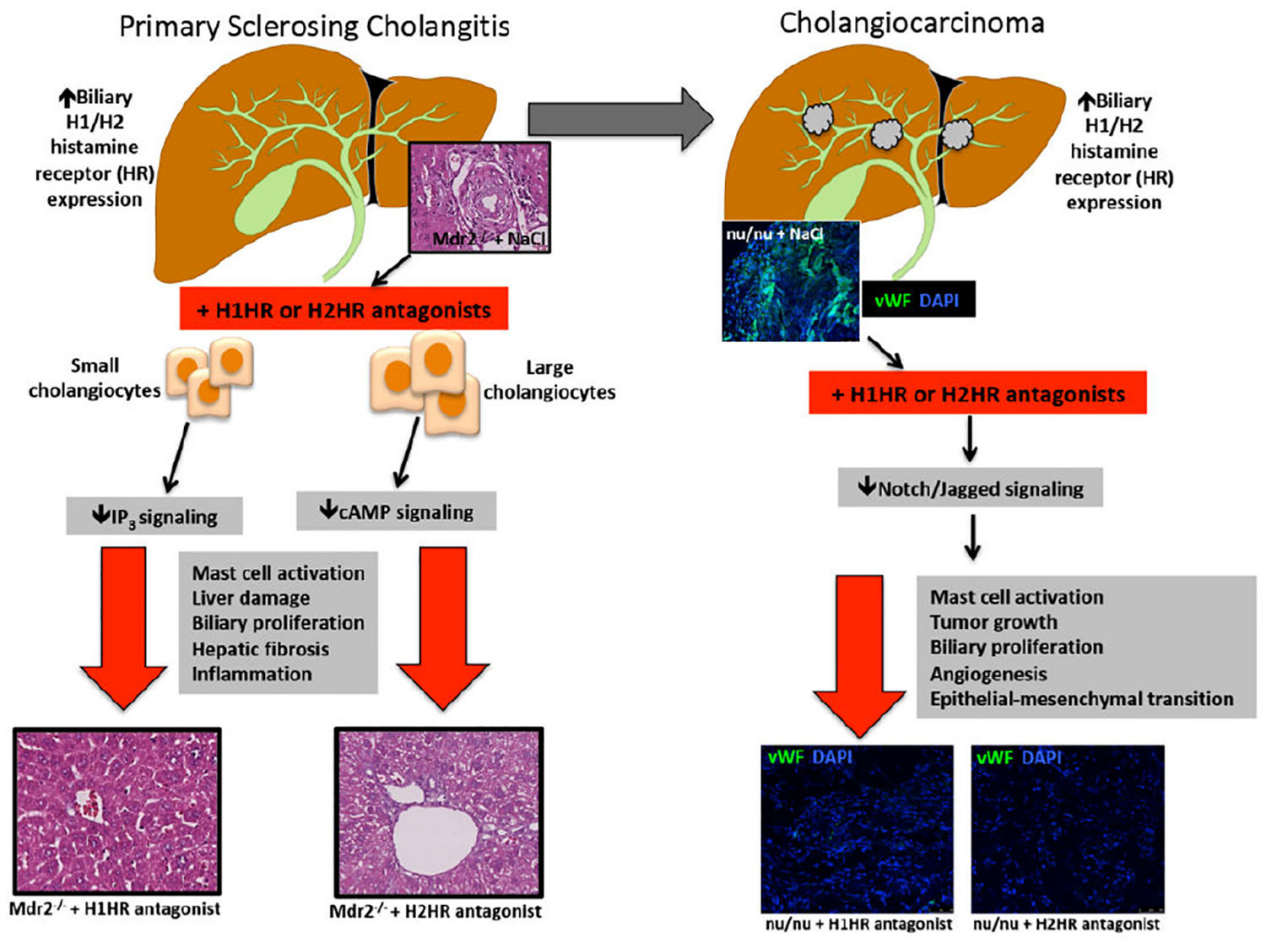
PSC can progress to cholangiocarcinoma (CCA). This study reveals that the H1 and H2 HRs are upregulated in both mouse models and human PSC and CCA. (LEFT) Blocking H1HR decreases small cholangiocyte proliferation via IP3 signaling, whereas H2HR decreases large cholangiocyte proliferation. Inhibition of either H1HR or H2HR or a combination of these drugs reduces overall liver damage, mast cell activation, biliary proliferation, hepatic fibrosis and inflammation. (RIGHT)Treatment with H1HR or H2HR inhibitors in nu/nu mice with xenograft CCA tumors results in decreased mast cell activation, tumor growth, biliary proliferation, angiogenesis and epithelial to mesenchymal transition via decreased Notch/Jagged signaling.

Mast cells have been indicated to play a role in many processes of hepatic injury [55,56]. Following hepatic injury, mast cells release migrates to the liver and is activated, thereby releasing increased histamine which stimulates biliary proliferation and hepatic fibrosis[57]. Mast cell migration is proportional to the progression of liver disease, and mast cells are found in close proximity to bile ducts following injury [55,57]. The multidrug resistance gene 2 (Mdr2) is an adenosine triphosphate-binding cassette transporter, which encodes a P-type glycoprotein responsible for the excretion of biliary phospholipids [58]. Damage in the livers of mice with the Mdr2 knocked out (Mdr2−/−) have been shown to mimic PSC-associated damage [59]. In Mdr2−/−, inhibition of mast cell-secreted histamine via cromolyn sodium treatment decreases biliary proliferation and hepatic fibrosis, highlighting the therapeutic potential for this compoun [58]. In a recent study by Kennedy et al. (Figure 2), the authors treated Mdr2−/− mice with over the counter antihistamines (blocking H1 histamine receptors (HR) or H2HR) for 4 weeks and found an amelioration of PSC-induced biliary damage, inflammation and hepatic fibrosis [60]. Mice were given treatments either separately or in combination and similar results were found [45]. These studies point to potential translational therapies for patients suffering from PSC since these drugs are readily available.

Immunosuppressive agents have traditionally not been shown to offer any mortality benefit in PSC. A meta-analysis and systemic review looked at several immunosuppressive agents in the treatment of PSC and found no survival benefit or decreased necessity of liver transplantation [61]. Conversely, four children with PSC were treated with mizoribine and azathioprine, immunosuppressants, and significant benefits were noted [62].

Two of the four children were treatment naïve patients and two had established liver cirrhosis. The treatment naïve patients were found to have normalization of liver enzymes following treatment and patients with established liver cirrhosis had improvement in liver histology as well as MRCP findings with the same treatment [62]. This combination therapy would need to be further studied with a much larger cohort to prove clinical significance. Additionally, a case report by Tischendorf et al. showed radiographic and laboratory improvement in a patient with PSC after a 13 month course of the immunosuppressant vedolizumab [63]. The patient had clinical improvement with reduction in pruritus and abdominal pain secondary to PSC. Success with immunosuppressant medications should call into question the diagnosis, as autoimmune cholangiopathy presents with overlapping features of PBC/PSC and autoimmune hepatitis [64,65].

## Biliary Atresia

### Introduction

Biliary atresia (BA) is characterized by fibrosis of the biliary tract in newborn infants that leads to obstruction of the extrahepatic bile ducts and subsequent liver cirrhosis indicative of liver transplant [66,67]. BA is a disease process that comprises anywhere from 34%−42% of neonatal jaundice [68]. The prevalence of BA was estimated to be 0.5 to 0.8 per 10,000 live births in Western countries [69]. The incidence of BA in the US was found to be 4.47 per 100,000 and was found to be higher in females (risk ratio of 1.43), and in Asian/Pacific Islander (risk ratio of 1.89) and African Americans (risk ratio of 1.30) as compared to Caucasians [70]. An infant suffering from BA would likely present with jaundice, polyspenia, vascular anomalies, situs inversus and cardiac abnormalities [71]. An ultrasound of the liver in patients suffering from BA would show echogenic fibrous tissue anterior to the portal vein, also known as the triangular cord sign [72]. The stool color card is the method that has gained the most attention due to its ease of implementation and non-invasive nature. The stool color card is the norm of diagnosis and contains photographs of normal and abnormal appearing stools. Use of the card, given to a postpartum mother, was found to reduce the amount of time that was taken before an infant was given treatment from approximately 70 days to 59.7 days. However, the card had a positive predictive value of only 12.7%, meaning that many infants that produced a positive test did not actually have BA [73]. The current treatment of BA is to re-establish bile flow via the use of the Kasai procedure, a portoenterostomy. This procedure, named after Dr. Morio Kasai, is preceded by the removal of the damaged bile ducts. Figure 1 describes the presentation, investigation and histological appearance of BA.

### Molecular mechanisms of injury

Studies have identified many factors in the pathogenesis of BA. In a 2014 study, a neonatal mouse model of rotavirus (RRV)-induced BA identified perforin and granzymes, released from NK cells and CD8+ T cells, may work synergistically to lead to cholangiocyte apoptosis [74]. When each of these molecular mechanisms was inhibited independently, cell lysis was miniscule. However, it was demonstrated that experimental BA could be successfully prevented by inhibition of both granules [74].

A 2013 human study used immunohistochemistry on resected bile ducts and liver biopsy specimens from 21 patients with BA (9 male/12 female; average age of 1.7 months) and compared them to specimens from one neonatal hepatitis patient and 6 patients with nonhepatobiliary diseases [75]. This study found that BA patients were found to have increased levels of interleukin-32 induced by biliary innate immunity via toll-like receptor 3 and other pro-inflammatory cytokines [75].

Studies that focused on the molecular mechanism of BA in mice have also found some promising information. In a 2017 study, TNFα and the expression of its receptors (TNFR1 or TNFR2) had been linked to apoptosis in bile duct epithelial cells [76]. The authors found that overexpression of TNFα by NK cells induced lysis of 55% ± 2% of cholangiocytes. Neonatal mice using the rotavirus (RRV)-induced BA model were given an antibody against TNFα, to block its downstream effects, or an antibody against TNFR1 or TNFR2 to block the binding of TNFα. The authors found that blocking TNFα or TNFR2 resulted in complete prevention of cholangiocyte lysis [76].

Histamine is a major inflammatory mediator that plays a role in cell proliferation, inflammation, extracellular matrix deposition and cell-matrix interactions [77]. Histamine has been linked to the migration of fibroblasts and the generation of hepatic fibrosis [77,78]. Finally, histamine has been found to play a role in the pathogenesis of BA. A recent study found that BA patients have increased hepatic histamine levels, which positively correlated with hepatic fibrosis. Additionally, hepatic l-histidine decarboxylase (HDC, synthesizes histamine) expression was increased, while monoamine oxidase B (MAOB, breaksdown histamine) was reduced in BA patients [79]. These findings corroborate what has previously been known about the profibrogenic role of hepatic histamine signalling [79].

### New therapeutic targets

Recently, surgeons have attempted to complete the previously discussed Kasai procedure in a laparoscopic manner [80]. A 2015 meta-analysis study compared an open portoenterostomy with the laparoscopic procedure and found that there was no statistically significant difference in operation time, hospital stay, intraoperative blood loss, cholangitis, or variceal bleeding between the two methods. Furthermore, 2-year survival (barring liver transplant) was higher in patients who elected for the open procedure [81]. On the contrary, Pediatric Surgery International published an article in which they reevaluated the minimally invasive surgical option. There were 22 cases of BA treated using the laparoscopic portoenterostomy between 2009 and 2016 which indicated that the use of this minimally invasive method resulted in the use of fewer anesthetics, less respiratory support and a minimal incidence of postoperative morbidities [82]. Since the advances of this procedure are new, it is clear that there will continue to be improvements in the logistics and quality of the operation.

In terms of BA prevention, a study involving the investigation of zebrafish who were exposed to factors that could potentially induce BA, found some groundbreaking news [83]. These fish were introduced to a previously unidentified isoflavonoid, biliatresone, 5 days after fertilization and were then studied for changes in bile ducts and gallbladders. This compound selectively destroyed extrahepatic bile ducts and was implicated in the destruction of cilia and disruption of cell polarity in neonatal mice. Furthermore, isofavonoids are currently found in many commonly eaten foods, such as chard, table beet, and sugar beet [83]. This is evidence that exposure to environmental factors during gestation does affect BA development, which could lead to promising actions that can be taken towards prevention of BA during the developmental stages of pregnancy.

Looking forward, there are many genes and proteins that may play a role in the pathophysiology of BA. GATA6 is a transcription factor that was found to be positively associated with cases of lung adenocarcinoma and is a possible target in the inhibition of progression of human laryngeal squamous cell carcinoma [84,85]. In terms of BA, GATA6 was found to have an elevated level of expression in cholangiocytes and hepatocytes in neonatal livers suffering from BA [86]. Additionally, completion of successful portoenterostomy downregulated the expression of GATA6 in these cells [86].

## Cholangiocarcinoma

### Introduction

**Figure 3:**
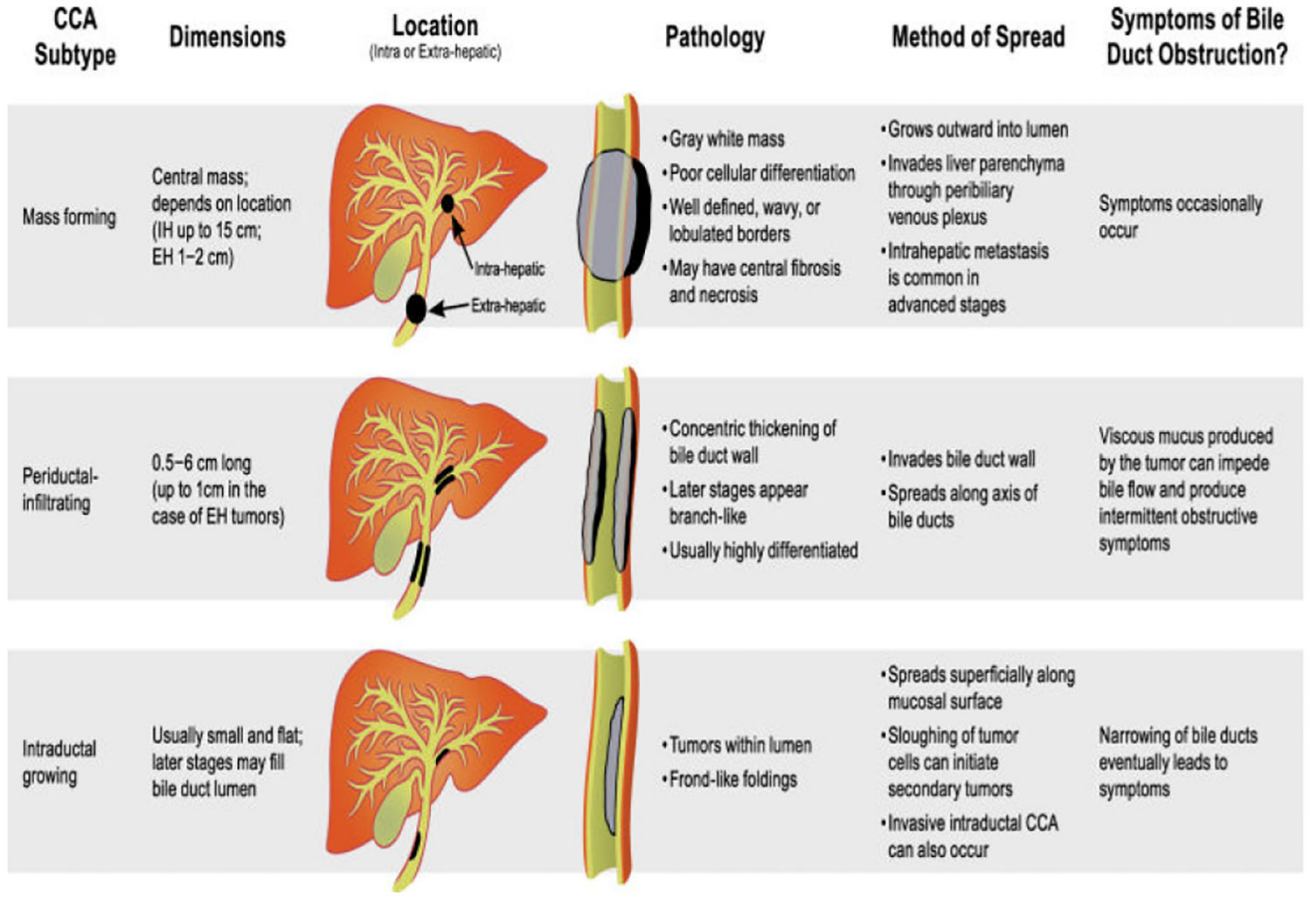
Characteristics of CCA including subtypes, mass formation, periductal infiltration/intraductal, location, pathology, symptoms and methods of metastasis. Adapted from Sripa et al. (2007). Liver fluke induces Cholangiocarcinoma. PLOS Medicine.

Cholangiocarcinoma (CCA) is a cancer that arises from the epithelial cells lining the bile ducts [87]. CCA is usually divided into two subtypes based on anatomical location: intrahepatic and extrahepatic. Within the extrahepatic category, there is perihilar and distal CCA [88]. A study including 564 patients between 1973 and 2004 found that the percentage of perihilar disease was approximated to be around 50%, while the percentage of distal CCA was found to be near 40%, leaving less than 10% of CCA cases to be classified as intrahepatic cases [89]. In terms of the incidence of these two subtypes of CCA in the United States, intrahepatic CCA has seen an increase in cases from 0.44–1.18 cases per 100,000 person-years between 1973 and 2012, whereas extrahepatic CCA has seen an increase in incidence from 0.96 to 1.02 per 100,000 over the same time period [90]. The symptoms that patients suffering from CCA present with depends on the type of cancer and its location. Some of the common symptoms include jaundice, pruritis, clay-colored stools, dark urine and other signs of biliary obstruction. The radiological findings present in patients suffering from CCA depend on the imaging modality and the growth pattern of the CCA. If the growth pattern was consistent with mass-forming intrahepatic CCA, an ultrasound would show a homogenous mass with a peripheral hypoechoic halo of compressed liver and the CT would show heterogenous material peripherally with central enhancement [91,92]. If the growth pattern was consistent with intraductal tumors, variability in biliary duct diameter and duct ectasia would be seen on CT and ultrasound 91. Figure 3 describes the characteristics of CCA including the subtype of CCA, size, location, pathology, metastatic potential, and symptoms.

Overall, primary liver cancer led to the second most cancer deaths in 2012 [93]. CCA is the second most common form of primary liver cancer, with hepatocellular carcinoma as the leading primary liver cancer [94]. It was reported that the highest rates of development of CCA were in Hispanic and Asian populations (1.8–3.3 per 100,000) with an increased prevalence in males (1.2–1.5 per 100,000) compared to females (1.0 per 100,00) [87]. Risk factors for developing CCA include PSC, fibropolycystic liver disease, parasitic infection, viral hepatitis, chronic liver disease, non-alcoholic fatty liver disease, obesity, type 2 diabetes mellitus and other genetic disorders [95,96]. A recent meta-analysis found that there was a moderate correlation between smoking and the development of CCA in Western countries (OR-1.35; CI-1.17 to 1.55) [88]. Another study stated that the increase in risk for developing intrahepatic and extrahepatic CCA was 46% and 77%, respectively [96]. Creating an environment in which cancer can grow is complex and involves many different changes. A recent article published in Human Pathology studied 54 intrahepatic tumors and found that 7.4% of cases were found to have mutated KRAS genes and a mutually exclusive 7.4% were found to have mutated BRAF genes[97]. Both of these genes are involved in the signalling of the RAS/MAPK pathway that is important for cell signalling and differentiation [97].

MicroRNAs are approximately 22 nucleotide long segments that regulate post-transcriptional regulation of gene expression. Specific microRNA segments have been identified to play different roles in the regulations of much pathology [98]. In a published study, the analysis of 80 different liver samples, half of which included tissues with intrahepatic CCA, identified that downregulated microRNA-26b-5p was associated with intrahepatic CCA diagnosis [99]. Additionally, downregulated microRNA-26b-5p correlated with increased invasive capacity of CCA. Administration of microRNA-26b-5p mimics reduced the invasion ability of CCA, and microRNA-26b-5p inhibitors were found to cause increased cell invasion ability. S100 calcium-binding protein A7, which is implicated in the progression of CCA, was found to be a downregulated by microRNA-26b-5p. This is the proposed target that causes the effect of decreased invasion of CCA [99]. MicroRNA-34a is another miRNA that has been found to play a role in the circadian rhythms of humans [100]. This miRNA targets Per1, which inhibits cell growth through various pathways and Per1 is decreased in CCA cells [100].

Inflammation and inflammatory signalling molecules pay an integral role in the development of CCA [101]. Interleukin 6 (IL-6) up regulates myeloid cell leukemia-1 (Mcl-1), a protein that is important in the anti-apoptotic factors in the development of cancer in the biliary epithelial cells [102]. IL-6 has been known to induce epithelial-tomesenchymal transition in CCA [103].

### New therapeutic targets

A 2018 study found that a MAPK inhibitor could be used in the treatment of lung cancers. The use of reactive oxygen species, such as hydrogen peroxide, was found to induce apoptosis in cancer cells. This could be translated to give new guidance in the treatment of CCA [104].

The effects of IL-6 have been implicated in the prevention of cell apoptosis that allows the development of CCA [97]. Although there are currently medications that are used for this purpose, there are many other drugs that are currently being assessed for safety and effectiveness. These medications are believed to reduce overall inflammation and may assist in alleviating some of the stress that is a precursor to the development of CCA. One such medication, Sarilumab, is an IL-6 receptor blocker that can be used to decrease the downstream effects of IL-6. This medication had demonstrated efficacy in one phase II and six phase III trials as of May 2017 [105].

One of the downstream effects of IL-6 is the upregulation of signal transducers and activators of transcription 3 (STAT3). It was found that activation of epidermal growth factor receptor (EGFR), fibroblast growth factor (FGFR), and platelet-derived growth factor receptor (PDGFR) via phosphorylation leads to activation of STAT3, and a subsequent increase of the cell cycle [106]. In 2009, Sorafenib, a multiple kinase inhibitor, was proven to downregulate the STAT3 pathway [107]. This drug has been known to inhibit EGFR and PDGFR. A 2017 study further analyzed this drug to find that the method of cell death has been found to be linked to cell cycle arrest between the G2 and M phases, leading to apoptosis and inhibition of growth. This was done via the downregulation of a regulatory protein and protein kinase, cyclin B1 and Cdk1, respectively [108].

Nitric oxide synthase is an enzyme found in endothelial cells that plays a role in vasodilation [109]. Interestingly, upregulation of nitric oxide synthase and its modulators has been shown to be correlated with increased angiogenesis and metastasis in CCA [110]. A study showed that inhibition of the nitric oxide synthase using nitro-L-arginine methyl ester hydrochloride (L-NAME) resulted in decreased migration and invasion of CCA, with a side effect of hypertension in treated patients [111]. This presents as another potential method for the prevention of CCA progression.

Histamine has been shown to exert pro-tumorigenic effects on CCA progression and angiogenesis [112] and several studies have demonstrated that manipulation of histamine receptors or the enzyme, histidine decarboxylase (HDC), regulates CCA tumor growth. Meng et al. showed that stimulation of H4HR decreased tumor growth both in vivo and in vitro [113] and another study demonstrated that blocking HDC using α-methyl-dl-histidine decreased CCA progression and angiogenesis [60]. Further, and more clinically relevant, a recent study by Kennedy et al. (Figure 2) demonstrated that in addition to blocking PSC [60], chronic antihistamine treatment, via H1HR or H2HR inhibition, prevented tumor progression and angiogenesis and reversed EMT in nu/nu mice. Importantly, histamine levels and HDC expression are increased in patients with CCA compared to controls, which might potentially be translated into biomarkers for early diagnosis.

## Conclusion

Given the fine interplay between molecular signalling molecules in the body and biliary tract cholestasis along with the fact that biological technical advances have reached rapid velocities, there will soon be much more information regarding cholangiopathies. By focusing on the molecular mechanisms highlighted in this article, there are many more potential therapeutic targets that need to be further evaluated. If these targets are appropriately vetted, it is possible that effective cures for these cholangiopathies may be discovered.

## Keypoints

Cholangiopathies are diseases that target cholangiocytes with limited therapeutic options/treatments. To date, liver transplantation is the only effective “cure” for cholangiopathies.

The molecular mechanisms regulating cholangiopathies has been widely investigated, yet there remains a lack of biomarkers and therapies.

Most cholangiopathies present at a later, progressed state of injury (except for Biliary Atresia, which is found shortly after birth) making patient outcomes dismal.

Translational studies are lacking and significantly warranted to uncover potential therapeutic avenues.

## Abbreviations

PBC: Primary biliary cholangitis
AMA: Antimitochondrial antibody
UDCA: Ursodeoxycholic acid
ALP: Alkaline phosphatase
AST: Aspartate transaminase
NK: Natural killer
E. coli: Escherichia coli
UC-MSC: Umbilical cord-derived mesenchymal stem cell
TE: Transient elastography
UC: Ulcerative colitis
PSC: Primary sclerosing cholangitis
IDO: Indoleamine 2,3 dioxygenase
TNF: Tumor necrosis factor
LSM: Liver stiffness measurement
IBD: Inflammatory bowel disease
MRCP: Magnetic resonance cholangiopancreatography
ERCP: Endoscopic retrograde cholangiopancreatography
PTC: Percutaneous trans-hepatic cholangiography
SASP: Senescence-associated secretory phenotype
TLR: Toll-like receptor
INF: Interferon
VAP-1: Vascular adhesion protein 1
MAdCAM-1: Mucosal addressin cell adhesion molecule 1
Mdr2: Multidrug resistance gene 2
Mdr2−/−: Mdr2 knock out
HR: Histamine receptors
BA: Biliary atresia
HDC-l: Histidine decarboxylase
MAOB: Monoamine oxidase B
CCA: Cholangio-carcinoma
IL-6: Interleukin 6
Mcl-1: Myeloid cell leukemia-1
STAT3: Signal transducers and activators of transcription 3
FGFR: Fibroblast growth factor
PDGFR: Platelet-derived growth factor receptor
L-NAME: Nitro-L-arginine methyl ester hydrochloride
HDC: Histidine decarboxylase
